# Analyzing bovine *OCT4* and *NANOG* enhancer activity in pluripotent stem cells using fluorescent protein reporters

**DOI:** 10.1371/journal.pone.0203923

**Published:** 2018-10-05

**Authors:** Delun Huang, Ling Wang, Neil C. Talbot, Chang Huang, Liping Pu, Xiuling Zhao, Xiuchun Tian, Ming Zhang, Young Tang

**Affiliations:** 1 State Key Laboratory for Conservation and Utilization of Subtropical Agro-Bioresources, Animal Reproduction Institute, Guangxi University, Nanning, Guangxi, China; 2 Department of Animal Science, Institute for Systems Genomics, University of Connecticut, Storrs, CT, United States of America; 3 U.S. Department of Agriculture, Agricultural Research Service, Animal Biosciences and Biotechnology Laboratory, Beltsville, MD, United States of America; The Roslin Institute, UNITED KINGDOM

## Abstract

Green fluorescent protein (GFP) reporters controlled by the regulatory region of *OCT4* and *NANOG*—two master regulators for pluripotency are widely used in studies of pluripotent stem cell establishment and embryo development. Alongside the challenge in establishing bovine pluripotent stem cells, the application of bovine-specific gene reporters has rarely been explored.

Using lentivirus-based GFP reporter, we investigated the upstream regulatory regions of bovine *OCT4* and *NANOG*. These reporters show activity in both naïve- and primed-state pluripotency when infected into mouse and human embryonic stem cells (ESCs), respectively. Consistent with what is found in humans and mice, the bovine *OCT4*-distal enhancer (b*OCT4*-DE) but not the proximal enhancer (b*OCT4*-PE) region is preferentially activated in naïve-state pluripotency. Furthermore, the b*OCT4*-DE region is silenced upon conversion of naive-state ESCs into primed-state epiblast stem cells (EpiSCs). Co-infection of mouse fibroblasts with the reprograming factors for induced pluripotent stem cell (iPSC) induction leads to the generation of GFP positive colonies, demonstrating that these GFP reporters can serve as live indicators for induced pluripotent cell establishment. We further proved that the bovine *OCT4* distal enhancer is active in bovine blastocysts. We established the lentiviral-based fluorescent reporters controlled by bovine *OCT4* and *NANOG* enhancer sequences. These reporter constructs show activity in naïve- and primed-pluripotent states. These reporters may serve as versatile tools for bovine ESC/iPSC generation and identification, as well as for developmental studies of bovine embryos.

## Introduction

Embryonic stem cells (ESCs) have infinite proliferation potential and are pluripotent. The induced pluripotent stem cells (iPSCs) resemble ESCs in pluripotency but are derived from somatic cells by overexpressing “reprogramming factors”, including *OCT4*, *KLF4*, *SOX2*, and *c-MYC* (OKSM), or *OCT4*, *SOX2*, *NANOG*, and *LIN28*[1, 2]. Mouse ESCs represent the pre-implantation stage “naïve” pluripotent state[3], with dome-shaped colony morphology, dependence on leukemia inhibitory factor (LIF) signaling for self-renewal, and their ability for single cell colonization[4, 5]. Conversely, post-implantation stage “primed” state pluripotency is represented by mouse epiblast stem cells (EpiSCs) and human ESCs, which are flat in colony morphology, dependent on basic fibroblast growth factor (bFGF)/Activin A signaling for pluripotency maintenance, and are refractory to single cell colonization[6–8]. Though both are considered pluripotent, the primed-state pluripotent stem cells exhibit limited chimera-forming capacity compared with cells of the naïve-state[6, 8, 9].

The naïve- or primed-state pluripotent ESCs/iPSCs derived from farm animals will be important cell resources for studies of livestock embryo development, animal reproduction, and human disease modeling. However, compared with other specie, ESC and iPSC development in bovine has been challenging. The effort of generating bovine ESCs started more than 21 years ago[10]. The reported putative bovine ESCs are very difficult to culture and usually differentiate within a few passages[11]. The limited propagation capacity is commonly seen in reported bovine iPSCs also[12–15]. These bovine iPSCs are not completely reprogrammed as shown by their dependence on the continued expression of the viral transgenes for self-renewal[12–15]. Also, there is a profound trophectoderm lineage generation or differentiation during bovine iPSC induction and ESC derivation[16, 17]. These problems negatively impact the practical applications of bovine ESCs/iPSCs. Tools are needed to help optimizing the conditions for bovine pluripotent stem cell development.

The use of green fluorescence protein (GFP) reporters controlled by the upstream regulatory regions of *OCT4* and *NANOG* has greatly facilitated mouse and human ESC/iPSC studies[18–20]. The transcription factors *OCT4* (also known as POU5F1) and *NANOG* are master pluripotency regulators essential for pluripotency maintenance and abundantly expressed in mammalian ESCs[21, 22]. They are also activated at the late reprogramming stage of iPSC induction[23]. The GFP reporters controlled by the enhancer regions of *OCT4* and *NANOG* offer non-invasive, convenient monitoring of the transcription activity for these genes to study pluripotency regulation and reprogramming kinetics in live cells. Also, in naïve-state pluripotency, the expression of human and mouse *OCT4* genes is preferentially regulated by their distal enhancers (*OCT4*-DE), which is silenced in primed-state mouse EpiSCs and human ESCs/iPSCs that preferentially use the *OCT4* proximal enhancer (*OCT4*-PE)[6, 24, 25] (S1 Fig). GFP reporters using this unique activity of *OCT4*-DE have been successfully applied to help generate naïve pluripotent human iPSCs[25–27].

Although the regulatory regions of *OCT4* and *NANOG* are highly conserved across humans, bovine, and mice[28, 29], study of the bovine-specific gene regulatory regions using fluorescent protein reporters has been scarce. A plasmid-based bovine *OCT4*-GFP reporter was shown to be active in mouse embryos[30]. Another plasmid-based GFP reporter controlled by a bovine *NANOG* regulatory fragment also showed activity in mouse ESCs[31]. However, plasmid-based reporters have low transfection efficiency and require lengthy selection and expansion for the creation of stably integrated cells. This can be cumbersome for iPSC induction that requires a large quantity of primary cells or secondary cells with low passage numbers (within P3-P5)[2, 32]. In this study, we utilized the high transduction efficiency of lentivirus-based GFP reporter vector to study the bovine-specific *OCT4* and *NANOG* regulatory regions. We show that these reporters are active in both mouse and human ESCs. We further show that the bovine *OCT4*-DE region is specifically activated in naïve-state pluripotent cells but not in primed-state pluripotent cells. We also tested these lentiviral reporters in mouse iPSC generation and show that they can serve as faithful indicators for pluripotency establishment. We also showed the activity of bovine *OCT4*-DE in bovine blastocysts from *in vitro* fertilization. The reporters we generated here will be valuable tools for the study of bovine ESC/iPSC generation, maintenance, regulation and differentiation, and in the study of bovine embryo development.

## Materials and methods

### Chemicals and constructs

Doxycycline (Dox) was purchased from Sigma-Aldrich (St. Louis, MO). PD0325901 and CHIR99021 were obtained from SelleckChem (Houston, TX). Polybrene was purchased from AmericanBIO (Natick, MA). Dox-inducible lentiviral FUW-TetO-hOKSM and FUW-M2rtTA plasmids were obtained from Addgene (Cambridge, MA). The human *OCT4* enhancer controlled pGreenFire Lenti-Reporter was from System Biosciences (Palo Alto, CA). Genomic DNA was extracted from bovine Wharton’s jelly cells using DNAzol (Invitrogen, Carlsbad, CA) according to the manufacturer’s protocol. Two bovine *OCT4* full-length enhancer fragments, two distal enhancers, one proximal enhancer and one *Nanog* enhancer were PCR amplified based on the published bovine *OCT4* gene sequence[28] and genome sequence Bos_Taurus_UMD3.1 from Ensemble[33]. The full-length *OCT4* enhancer fragments were designated as b*OCT4*-GFP1 (-2851 to -1 bp relative to the ATG start codon) and b*OCT4*-GFP2 (-3851 to -1 bp, see GeneBank access number MF664108 for sequence information of bovine OCT4), respectively.

For the *OCT4* distal enhancer region, b*Oct4*-DE was from -3,851 to -1644 bp and b*OCT4*-DE2 was from -3,851 to -1644bp plus the *OCT4* minimal promoter (-260 to -1 bp). The *OCT4* proximal enhancer was amplified from -1645 to -1 bp. The *Nanog* enhancer was cloned from -1096 bp to -1 bp. For cloning primer sequence information see S1 Table. All the fragments were inserted into the pGreenFire Lenti_Reporter Vector (System Biosciences) using the In-Fusion kit (Clontech, Mountain View, CA). All the DNA constructs were verified by direct DNA sequencing.

### Lentivirus preparation

The verified lentiviral b*OCT4* or b*NANOG* reporter constructs, the FUW-TetO-hOKSM or FUW-M2rtTA were co-transfected with the lentiviral packaging plasmids pCMV-V-SVG and psPAX2 (Addgene, Cambridge, CA) into 293T cells using Fugene 6 reagent (Promega, Madison, WI) based on the viral packaging protocol provided from Addgene.com. The virus-containing supernatant was harvested 48 h and 72 h post-transfection and filtered with 0.8 μm sterile filters (Corning Inc., Corning, NY).

### Cell culture, viral transduction and mouse embryonic fibroblast (MEF) reprogramming

Mouse R1-ESCs were cultured in 2i/LIF medium[34] on mitomycin C-treated MEF feeders. Human H9-ESCs were cultured in feeder-free mTeSR1 (Stemcell Technologies, Cambridge, MA) medium on matrigel (Corning). MEFs were cultured in medium consisting of DMEM (Invitrogen) with 10% fetal bovine serum (FBS) (Rocky Mountain Biologicals, Inc., Missoula, MT). Lentiviruses of different reporters were transduced into mouse or human ESCs with polybrene. For transduction of R1-ESCs, the next day after seeded as single cells by trypsinization, the cells were incubated with viruses and polybrene containing medium overnight. For transduction of human ESCs, cells were digested with 1 mg/ml Dispase into cell aggregates and seeded on day 0. Two lentiviral infections were performed on day 1 and day 2, respectively. The mouse and human ESCs were then maintained in their respective medium for the appearance of GFP signal after transduction.

For reprogramming, 1 million B6/129 MEFs at passage 2 were seeded on a gelatin coated six-well-plate on day -1. On day 0, lentiviruses of different reporters were co-transduced into MEFs together with the Dox-inducible polycistronic FUW-TetO-hOKSM and FUW-M2rtTA in the presence of polybrene. On day 1, the medium was replaced to MEF medium with 1μg/ml Dox. The infected MEFs were passaged onto MEF-feeders on day 2. Starting from day 3, reprogramming medium containing 1μg/ml Dox was used for continued reprogramming. The reprogramming medium was a 1:1 mixture of the KSR-ESC medium containing 20% Knock-out serum replacement (KSR), 1 x MEM non-essential amino acids, 1 x GlutaMax, 0.5 x penicillin/streptomycin, 1 x 2-mercaptoethanol and 1,000 U/mL mLIF in Knock-out DMEM (all from Invitrogen), and the serum-containing ES medium containing 20% ES-FBS, 1 x MEM non-essential amino acids, 1 x GlutaMax, 0.5 x penicillin/streptomycin, 1 x 2-mercaptoethanol and 1,000 U/ml mLIF in DMEM (all from Invitrogen). The GFP positive mouse iPSC colonies were picked and expanded in 2i/LIF medium without Dox.

### Embryoid body (EB) formation

The mouse ESCs carrying reporters were dissociated with 0.05% trypsin (Invitrogen), and then plated into 60 mm tissue culture dishes for 20 min to allow MEF-feeders to attach. The ESCs remaining in the medium were transferred to 60 mm low adhesive Petri dishes for differentiation in DMEM (Invitrogen) plus 10% FBS (Rocky Mountain Biologicals, Inc.) without LIF. After 1 wk of differentiation, EBs were either subjected to RNA extraction or transferred to cell culture dishes coated with 0.1% gelatin (Merck Millipore, Billerica, MA) for further differentiation before being subjected to RNA extraction.

### Epiblast stem cell (EpiSC) differentiation

EpiSC differentiation was performed based on a previous study[35]. Briefly, mouse R1-ESCs infected with reporters were seeded at a density of 1×10^5^ per well in a 6-well plate in which MEF-feeders had been plated beforehand. Twenty-four hours after plating, the medium was changed to EpiSC medium which consists of DMEM-F12, 15% KSR, 1 x GlutaMax, 1 x MEM non-essential amino acids (all from Invitrogen), 1 x 2-mercaptoethanol (Merck Millipore, MA), 12 ng/mL FGF2 (R&D Systems, Minneapolis, MN), 20 ng/mL Activin A (R&D Systems) and 1 x penicillin/streptomycin. Thereafter, cells were maintained in EpiSC medium and passaged every 3–4 days using 1 mg/mL collagenase type IV. After 4 passages, EpiSC colonies were used for flow cytometry and other analyses.

### Immunostaining

Alkaline phosphatase staining was performed with the Vector Red Alkaline Phosphate Substrate Kit (Vector Laboratories, Burlingame, CA). For immunostaining, cells were fixed in 4% paraformaldehyde with 1% sucrose in phosphate buffered saline (PBS) (Invitrogen) for 15 min at room temperature. The cell membranes were then permeabilized with 0.5% TX-100 in PBS-T (Sigma-Aldrich), blocked in donkey serum (Sigma-Aldrich) and incubated with donkey serum containing primary antibodies including rabbit anti-*OCT4* (Merck Millipore), rabbit anti-SOX2 (Abcam, San Francisco, CA) and rabbit anti-NANOG (Merck Millipore) at 1:100 dilutions, washed in PBS-T, and then incubated with Alexa Fluor 594 conjugated goat anti-rabbit secondary antibody (Cell Signaling Technology, Danvers, MA) at a 1:500 dilution. The cell nuclei were counterstained with DAPI. Fluorescent images were taken using a Zeiss or Nikon fluorescence microscope.

### Fluorescence flow cytometry analysis

The infected mouse R1-ES cells, EpiSCs, and EBs for flow cytometry analysis were dissociated with 0.05% trypsin and resuspended in PBS containing 2% FBS. 10,000 events of cell counts were analyzed for each sample. The dead cells were distinguished with propidium iodide (PI) staining. The live cell populations were analyzed for their GFP fluorescence intensity. A BD Accuri C6 Flow Cytometer (BD Biosciences, San Jose, CA) and FlowJo software were used for the measurement and data analysis, respectively.

### Quantitative real time—PCR (qRT-PCR)

Total RNA was extracted with Trizol (Invitrogen) based on the manufacturer’s instruction. One μg of the total RNA was reverse-transcribed into cDNA with an All-In-One cDNA Synthesis kit (Bimake, Houston, TX). Real-time PCR reactions were performed with the SYBR Green qPCR Master Mix (Bimake) and an ABI 7500 Fast platform (Thermo Fisher Scientific). The 7500 software v2.3 was used for data analysis, with values normalized to GAPDH.

### In-vitro fertilization (IVF) and cytoplasmic injection

Oocytes were collected from *Bubalus bubalis* ovaries obtained from a local slaughter house. All procedures involving animal treatment and collection of ovaries in the study were based on the Guiding Principles for animal use as described by the Council for Institutional Animal Care and Use Committee (IACUC) and approved by the Animal Experimentation Ethics Committee of Guangxi University, Nanning, China. The IVF procedure was performed as described previously[36]. Briefly, IVF were performed after oocyte maturation for 24 hours in the medium containing TCM-199, 10% Oestrus Calf Serum (OCS), 0.5 mg/mL Follicle-Stimulating Hormone (FSH), 5 mg/mL Luteinizing Hormone (LH) and 1 mg/mL estradiol-17β at 38.5°C under 5% CO2 atmosphere with maximum relative humidity. Following IVF, the presumptive zygotes were cultured in IVF medium (modified Tyrode's medium supplemented with 36% TCM-199, 10% fetal bovine serum, 0.06 mg/ml penicillin and 0.1 mg/mL streptomycin) at 38.5°C under 5% CO2 atmosphere with maximum relative humidity. Cytoplasmic injection was carried out 7 hours after IVF with IVF medium containing 0.02μl/ml cytochalasin-b with a Nikon ECLIPSE Ti micromanipulator. The reporter plasmid concentration was 30ng/μl. After cytoplasmic injection, the embryos were monitored daily with an OLYMPUS DP73 fluorescent microscope until hatching.

### Statistics

Each experiment was repeated at least three times (n ≥ 3). Quantification of GFP fluorescence intensity from flow cytometry analysis and the relative gene expression from qRT-PCR analysis was presented as the mean ± SD (standard deviation). Data were processed using One-Way ANOVA with Tukey's multiple comparisons, or the Student’s T-test. A p-value < 0.05 was considered statistically significant.

## Results

### Reporters controlled by bovine *OCT4* and *NANOG* enhancers are active in primed- and naïve-state pluripotent stem cells

**Fig 1:**
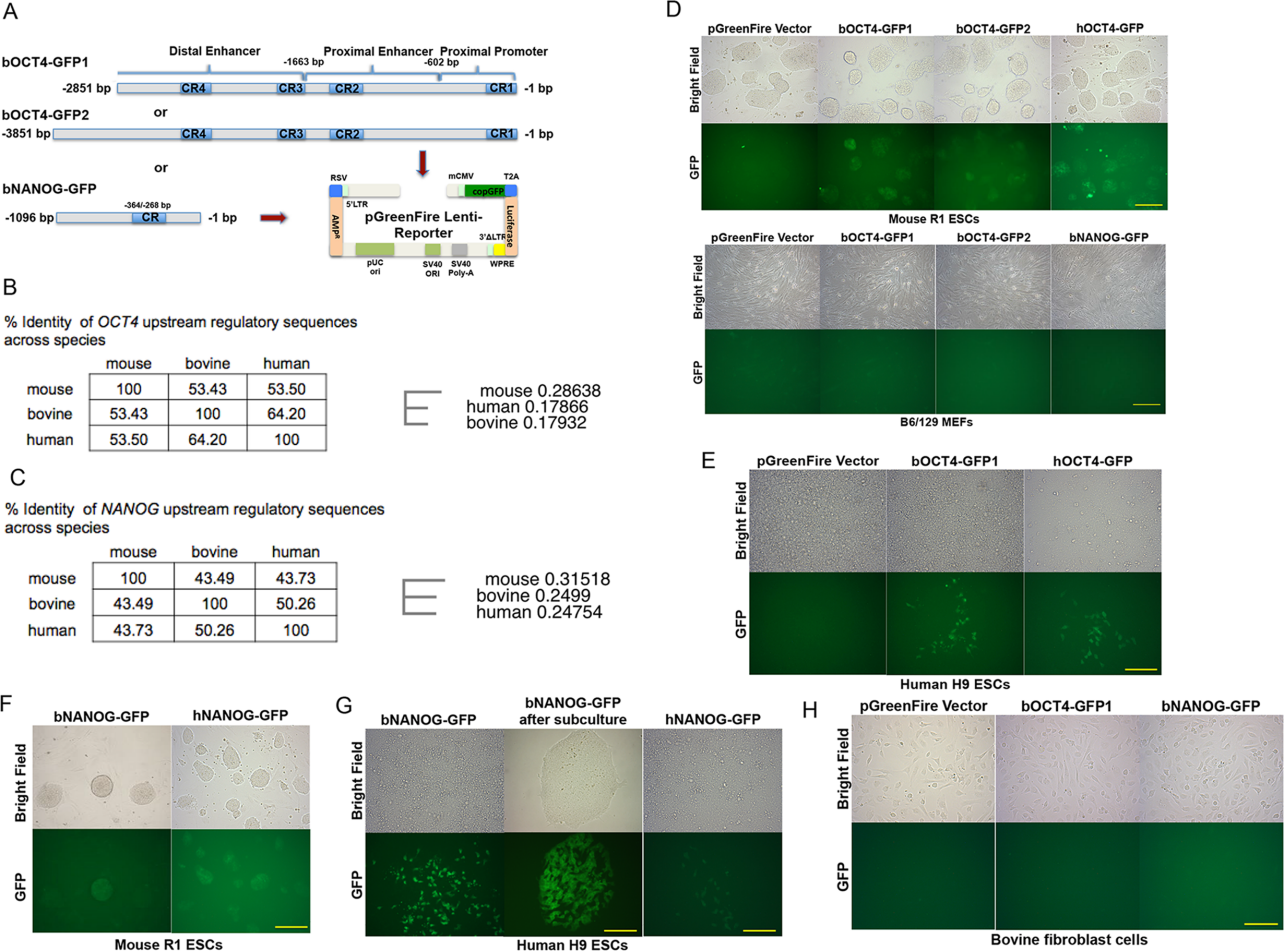
Bovine *OCT4* and *NANOG* enhancers active in naive and primed pluripotent stem cells. A: Schematic representation of bovine *OCT4* and *NANOG* (b*OCT4*, b*NANOG*) regulatory regions upstream of ATG start codon and cloning into the pGreenFire Lenti-Reporter. The conserved-regions (CR) of *OCT4* and *NANOG* regulatory regions are also shown. The b*OCT4* distal and proximal enhancers and proximal promoter are shown based on sequence homology to the human and mouse *OCT4*. B. Sequence homology (left) and phylogenetic tree (right) of OCT4 upstream regulatory regions. Multiple sequence alignment was performed with Clustal Omega. C. Sequence homology (left) and phylogenetic tree (right) of NANOG upstream regulatory regions. Multiple sequence alignment was performed with Clustal Omega. D: Upper: b*OCT4*-GFP lentiviral reporters transduced into single cell mouse R1 ESCs and cultured for 7 days in 2i/LIF medium. pGreenFire reporter vector and the human *OCT4* enhancer controlled GFP reporter (h*OCT4*-GFP) were used as negative and positive controls, respectively. Lower: b*OCT4*- and b*NANOG*-GFP lentiviruses infected into MEFs for 7 days. pGreenFire reporter vector was used as control. Bar = 250 μm. E: b*OCT4*-GFP lentivirus was infected into human H9 ESCs and cultured for 7 d. pGreenFire reporter vector and the h*OCT4*-GFP reporter were used as negative and positive controls, respectively. Bar = 250 μm. F: b*NANOG*-GFP lentiviral reporter expresses in single cells of mouse R1 ESCs and cultured for 7 days in 2i/LIF medium. The human *NANOG* enhancer controlled GFP reporter (h*NANOG*-GFP) was used as a positive control. Bar = 250 μm. G: Left: b*NANOG*-GFP lentivirus was infected into human H9 ESCs and cultured for 7 d. Middle: enrichment of positive b*NANOG*-GFP expressing cells in H9 ESC colonies after passaging (P8). Right: h*NANOG*-GFP lentivirus used as a positive control. Bar = 250 μm. H. b*OCT4*- and b*NANOG*-GFP lentiviruses infected into bovine fibroblast cells. pGreenFire reporter vector was used as control. Bar = 250 μm.

The ~2.9 kb bovine *OCT4* gene 5’ upstream regulatory region[28] share 53.43% and 64.2% sequence identity with mouse and human *OCT4* upstream regulatory regions, respectively. In addition, this upstream regulatory region contains four highly conserved regions (CR1-4)(Fig 1A and 1B) across the three species[29]. To investigate the bovine *OCT4* enhancer activity, we cloned the bovine *OCT4* upstream regulatory regions comprising 2,851 and 3,851 nucleotides from the translational start codon into the lentiviral pGreenFire Lenti-Reporter (b*OCT4*-GFP1 and–GFP2, respectively, Fig 1A). We first asked whether these reporters would work in the naïve pluripotent mouse and primed pluripotent human ESCs, respectively. Transduction of the lentiviral b*OCT4*-GFP to single cells of mouse R1-ESCs yielded a high percentage of GFP positive (GFP+) colonies after one wk in culture in 2i/LIF medium, known to be restrictive to ground state pluripotency[34]. No GFP+ colony was observed in control vector infection (Fig 1D). Infection of the b*OCT4*-GFP lentivirus into mouse embryonic fibroblasts (MEFs) generated no GFP+ signal (Fig 1D). These results indicated that the bovine *OCT4*-enhancer could be activated in naïve pluripotent mouse ESCs. We then infected the lentiviral b*OCT4*-GFP into human H9 ESCs. These primed-state ESCs can only be passaged through incomplete digestion into small clumps of cells, therefore the infection efficiency was not high. Nevertheless, we observed scattered GFP+ cells within individual colonies at one wk after infection (Fig 1E).

We also cloned a 1,095 nucleotide bovine *NANOG* 5’ regulatory region, based on a previous study[31], into the pGreenFire Lenti-Reporter (b*NANOG*-GFP, Fig 1A). This bovine *NANOG* upstream regulatory region shares 43.49% and 50.26% sequence identity with mouse and human *NANOG* regulatory regions, respectively (Fig 1C). The bovine *NANOG* regulatory region also contains an across-species conserved region[31], as shown in Fig 1A. We observed activation of this reporter in mouse ESCs (Fig 1F), and, similar to our results with b*OCT4*-GFP, scattered GFP+ cells in individual colonies upon infection into human H9 ESCs, which could be further enriched through subculturing (Fig 1G). We also transduced these bovine *OCT4*- and *NANOG*-GFP lentiviral reporters into bovine fibroblast cells, no fluorescence was observed (Fig 1H). Thus, these bovine *OCT4*- and *NANOG*-GFP lentiviral reporters were specifically active in both naïve- and primed-state pluripotent ESCs.

### Bovine *OCT4* distal and proximal enhancers exhibit different activities in naïve pluripotent stem cells

**Fig 2:**
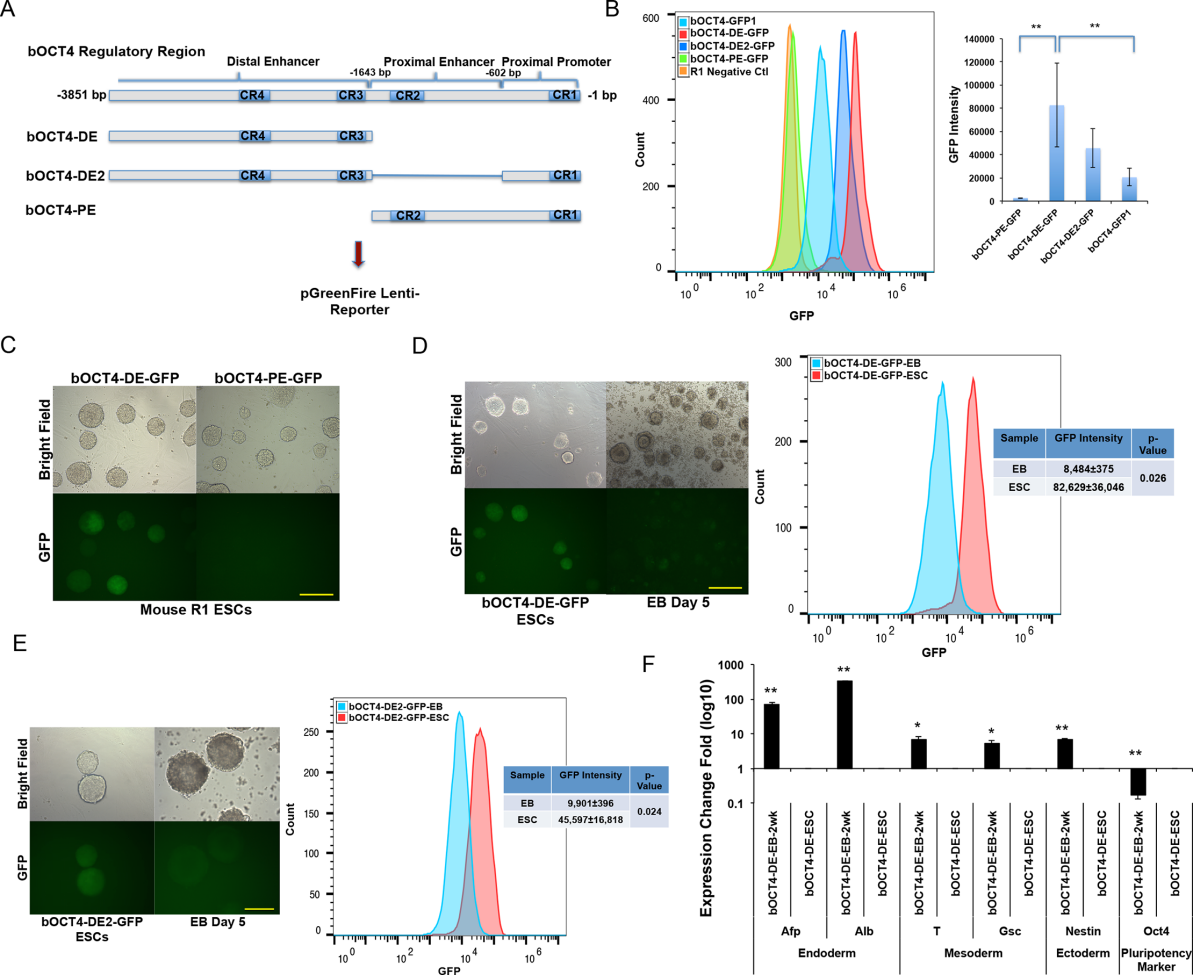
Bovine *OCT4* distal and proximal enhancers act differently in naïve pluripotent stem cells. A: Schematic representation of bovine OCT4 proximal and distal regulatory regions upstream of ATG start codon cloning into the pGreenFire Lenti-Reporter. B: Left: Fluorescence flow cytometry analysis of b*OCT4*-DE-GFP, b*OCT4*-DE2-GFP, b*OCT4*-GFP1, and b*OCT4*-PE-GFP expression in R1-ESCs. Non-infected R1-ESCs served as the negative control (Ctl). Right: GFP intensity measured by flow cytometry. Bars represent mean±sd, **: p < 0.01, n = 4.C: Expression of b*OCT4*-DE-GFP and b*OCT4*-PE-GFP in R1-ESCs. Bar = 250 μm.D: Left: Cloned b*OCT4*-DE-GFP positive ESCs and the EBs formed at 1-wk upon differentiation. Bar = 250 μm. Right: Fluorescence flow cytometry analysis of b*OCT4*-DE-GFP positive ESCs and their differentiated cells at 1-wk of differentiation. GFP intensity represents mean±sd, n = 4.E: Left: Cloned b*OCT4*-DE2-GFP positive ESCs and the EBs formed at 1-wk upon differentiation. Bar = 250 μm. Right: Fluorescence flow cytometry analysis of b*OCT4*-DE2-GFP positive ESCs and the differentiated cells at 1-wk of differentiation. GFP intensity represents mean±sd, n = 3.F: qRT-PCR analysis of endogenous *Oct4* and differentiation marker genes at 1-wk of EB differentiation from the b*OCT4*-DE-GFP+ ESCs. Values are normalized to GAPDH and compared with the undifferentiated ESCs. Bars represent mean±sd, **: p < 0.01, n = 3.

The human *OCT4*-DE (from CR3 to ~3.8 kb upstream of the translation start site) is preferentially activated in the naïve-state, while the *OCT4*-PE (nucleotides -1,835 to -681 bp) is activated in primed-state pluripotent cells[25, 26]. Based on the high sequence homology between human and bovine *OCT4*, we asked if the bovine *OCT4* DE and PE regions (b*OCT4*-DE and b*OCT4*-DE) would act in a similar way. We removed the bovine *OCT4* PE region (nucleotides -1,643- to -602 bp) out of the 3,851 nucleotides b*OCT4* regulatory sequence (b*OCT4*-DE), or with an additional deletion of the proximal promoter (nucleotides -601 to -1) (b*OCT4*–DE2), based on the sequence homology between bovine and human *OCT4*[29] (Fig 2A). We cloned these b*OCT4* regulatory regions into the pGreenFire Lenti-Reporter and evaluated their activity in mouse R1-ESCs. Fluorescence flow cytometry analysis and microscopy observation revealed that both b*OCT4*-DE and–DE2 fragments were active in naïve-state ESCs, while the b*OCT4*-PE was not (Fig 2B and 2C). Interestingly, the b*OCT4*-DE-GFP showed significantly stronger GFP intensity than that of b*OCT4*-GFP1 in R1-ESCs (Fig 2B). Thus, similar to their human and mouse *OCT4* counterparts, the bovine *OCT4* distal, but not proximal regulatory region, was active in naïve-state pluripotent ESCs.

We then asked whether the b*OCT4*-DE-GFP reporter would be silenced after the differentiation of R1-ESCs. We observed marked decrease in GFP expression after 1-wk of EB differentiation upon LIF removal from the mouse ESCs (Fig 2D). The decrease in GFP intensity was confirmed by flow cytometry analysis (Fig 2D). The case was similar for the EB differentiation from b*OCT4*-DE2-GFP+ mouse ESCs (Fig 2E). qRT-PCR analysis further verified that the decreased GFP expression correlates with a reduction of endogenous *Oct4* expression and increased expression of commitment genes in the differentiated cells (Fig 2F).

### Bovine *OCT4* distal enhancer is silenced in primed-state pluripotent stem cells

**Fig 3:**
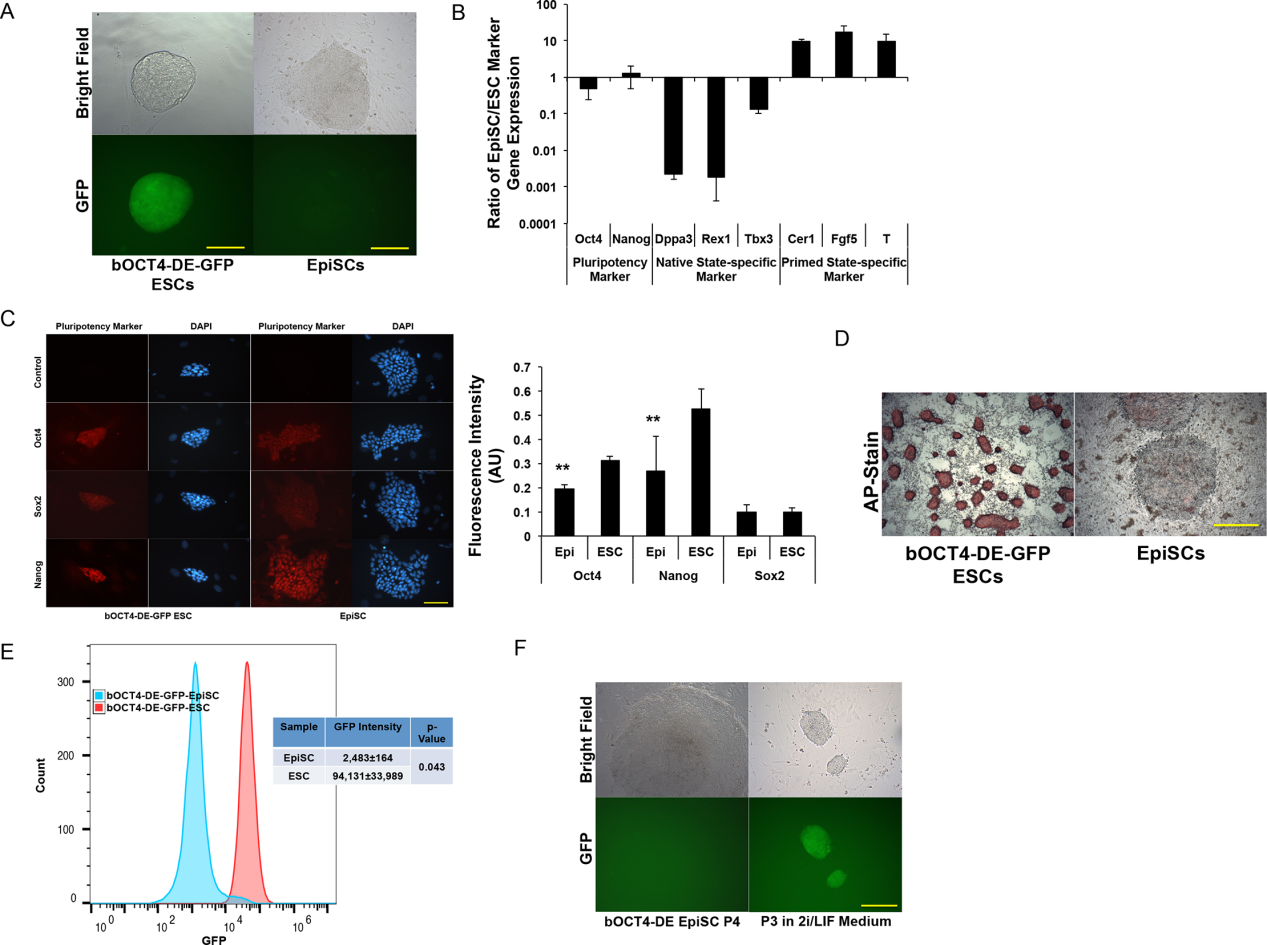
Bovine *OCT4* distal enhancer silenced in primed pluripotent stem cells. A: The parental b*OCT4*-DE-GFP+ ESCs and their EpiSC colonies formed after subculturing (P4) in EpiSC medium. Bar = 120 μm (left) or 120 μm (right).B: qRT-PCR gene expression ratio for primed- and naïve-state pluripotency markers between the converted EpiSCs (p4) and their parent ESCs. n = 3.C: Left: Immunostaining of pluripotent markers in ESCs and the converted EpiSCs. Bar = 60 μm. Right: Quantification of fluorescence intensity from immunostaining. Values are arbitrary unit (AU) normalized with DAPI Stain. **: p < 0.01, n = 5.D: AP-staining of EpiSCs and their parental ESC colonies. Bar = 250 μm.E: Fluorescence flow cytometry analysis of b*OCT4*-DE-GFP positive ESCs and their converted EpiSCs (p4). GFP intensity represents mean±sd, n = 3.F: b*OCT4*-DE-GFP negative EpiSC colonies (left) were further expanded and developed into GFP+ ESC-like colonies (right) upon culture in 2i/LIF medium and passaged for 3 times (P3). Bar = 250 μm.

We further asked whether the b*OCT4* distal enhancer region would be silenced in primed pluripotent state cells. We converted the b*OCT4*-DE-GFP+ naïve-state R1-ESCs to primed-state EpiSCs by culturing them in Activin A and bFGF containing medium[35]. Upon 2–3 passages, these cells formed flat monolayer EpiSC-like colonies resembling the typical primed-pluripotent state morphology, and they exhibited diminished GFP expression (Fig 3A). Compared with their parental naïve-state ESCs, these cells displayed markedly suppressed expression of naïve-specific markers (Dppa3, Rex1, Tbx3), and an increased profile of EpiSC-specific markers including Cer1, Fgf5, and T (Fig 3B). The expression of pluripotency markers including *Oct4*, *Nanog*, and *Sox2* remained similar or slightly lower in EpiSCs than their parental ESCs (Fig 3B and 3C). Unlike the naïve-state ESCs, EpiSCs exhibit little alkaline phosphatase (AP) activity[8]. This was also demonstrated in these converted EpiSCs (Fig 3D). The decreased b*OCT4*-DE-GFP expression in these EpiSCs was further confirmed with fluorescence flow cytometry (Fig 3E). Thus, the bovine *OCT4* distal enhancer was inactivated in primed-state pluripotency.

Primed-state EpiSCs can be successfully converted to naïve-state ESCs when directly cultured in the naïve-selective 2i/LIF medium, although at low efficiency[37–39]. We wondered whether the GFP-negative EpiSCs we generated here could also be converted back to ESC-like cells and become GFP+ again. To avoid any remaining naïve GFP+ cells in the EpiSC culture, single GFP- negative EpiSC colonies were picked and expanded further through passaging. After EpiSC expansion, we cultured the expanded b*OCT4*-DE-GFP negative EpiSC colonies in 2i/LIF medium. Significant cell death and differentiation occurred to these cells upon switching to 2i/LIF medium similarly as described[35]. However, after 3 passages, we observed the emergence of several colonies with typical dome-shaped naïve ESC-like morphology, which were GFP+ (Fig 3F). Thus, the bovine *OCT4* distal enhancer is specifically activated in naïve-state pluripotent cells.

### Reporters controlled by bovine *OCT4* enhancer are activated during somatic cell reprogramming

**Fig 4:**
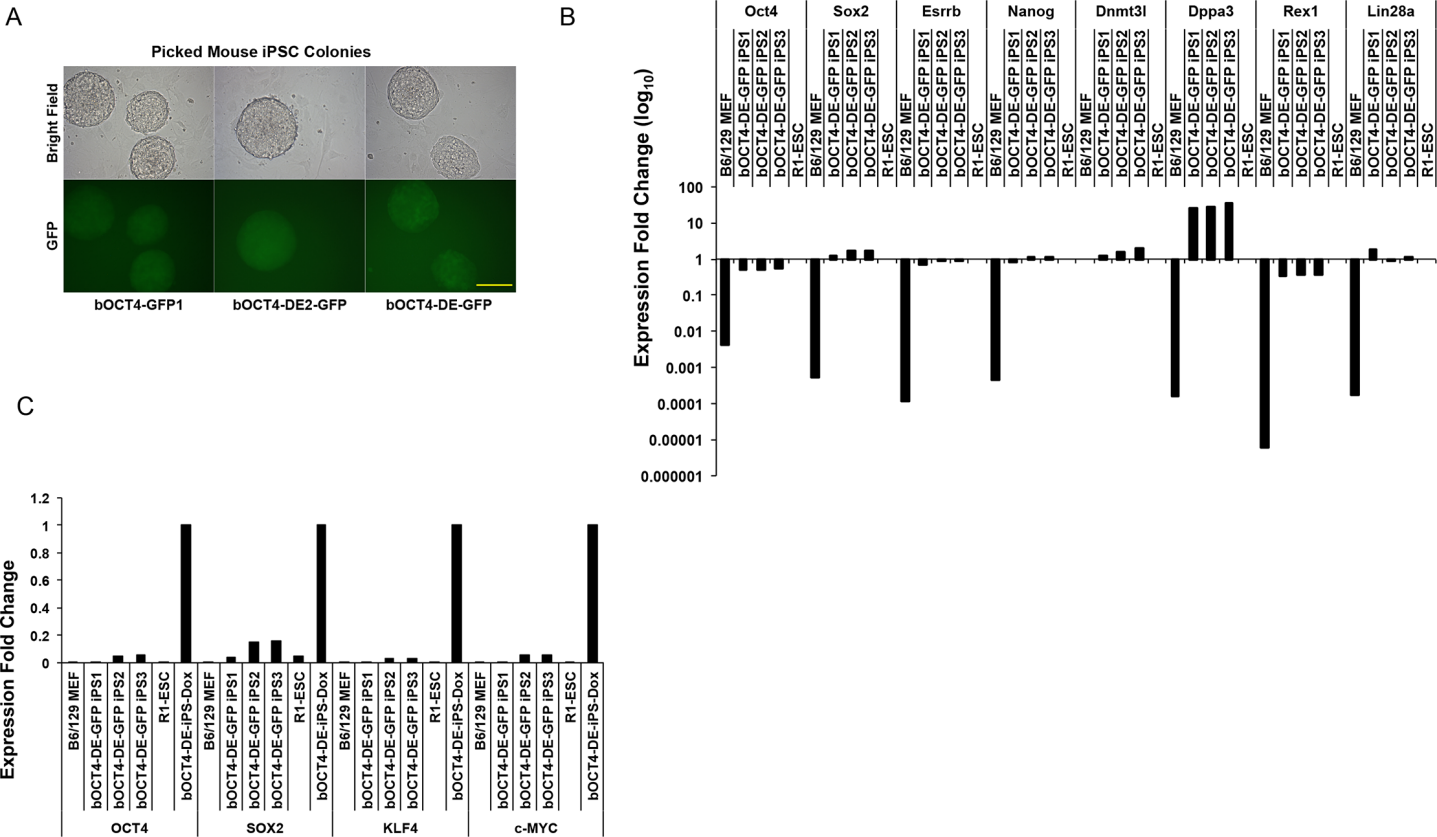
Bovine *OCT4* enhancer GFP-reporters activated during somatic cell reprogramming process. A: Picked GFP+ putative iPSC colonies infected with b*OCT4*-GFP1, b*OCT4*-DE2-GFP, and b*OCT4*-DE-GFP and cultured in DOX-free ESC culture medium (P2). Bar = 120 μm.B: qRT-PCR for endogenous pluripotent gene expression from three expanded iPSC lines positive for b*OCT4*-DE-GFP (P2). R1-ESCs and MEFs were used as the positive and negative control, respectively. C: qRT-PCR for transgene expression from three expanded iPSC lines positive for b*OCT4*-DE-GFP (P2). R1-ESCs and MEFs were used as negative controls, and b*OCT4*-DE-GFP positive iPSCs cultured in Dox-containing medium are used as positive control.

We wondered whether the b*OCT4*-GFP reporters we generated might be used as live indicators in reprogramming cells for iPSC generation. To test this we used the MEF reprogramming system with doxycycline (Dox)-inducible OKSM expression[40] as bovine iPSC generation remains a challenge[16]. B6/129 MEFs were infected with lentiviral OKSM plus lentivirus of empty pGreenFire vector, b*OCT4*-GFP1, b*OCT4*-DE-GFP, or b*OCT4*-DE2-GFP. The infected MEFs were then passaged onto CD1-feeders and cultured in reprogramming medium. We started to observe GFP fluorescence on day 11 after viral infection from colonies of b*OCT4*-GFP1, b*OCT4*-DE-GFP, and b*OCT4*-DE2-GFP, but not the control vector reporter infected reprogramming conditions. We picked the GFP+ colonies carrying different reporters at reprogramming wk 2 to 3. The picked colonies can be cultured in Dox-free ESC culture medium and remain GFP positive (Fig 4A). We further expanded the *OCT4*-DE-GFP colonies and qRT-PCR analysis showed that these cells had significantly activated endogenous pluripotent genes such as *Oct4*, *Nanog*, *Sox2*, and *Rex1*, etc., that are comparable to R1-ESCs (Fig 4B). Furthermore, these iPSC colonies showed silenced transgene expression compared with those still remained in Dox-containing medium (Fig 4C). Thus, our results indicate that these bovine-specific *OCT4*-GFP reporters can be used as real-time indicators for successful reprogramming.

### Bovine *OCT4* distal enhancer is active in bovine blastocysts

**Fig 5:**
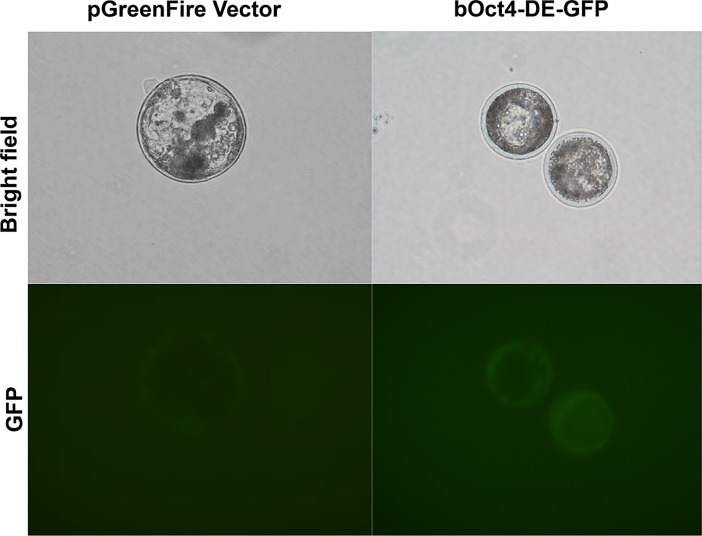
Bovine *OCT4* distal enhancer is active in bovine day 9 blastocyst from in vitro fertilization. pGreenFire reporter vector was used as a negative control.

To test if our bovine OCT4-DE-GFP reporter constructs can be activated in bovine embryonic-stage cells, we microinjected the plasmids (GFP Control and bovine *OCT4*-DE-GFP vectors) into bovine *in vitro* fertilized (IVF) zygotes. Despite the challenges that only cytoplasmic injection was performed due to the invisibility of bovine pronuclei in the zygotes and that blastocyst development rate are low (~ 10%) after plasmid DNA injection, we observed GFP fluorescence in day 9 bovine blastocyst cells injected with bovine *OCT4*-DE-GFP (Fig 5). Interestingly, this reporter is active in both the inner cell mass (ICM) cells and trophoblast cells in the bovine embryos (Fig 5). This agrees with the previously report that mouse *Oct4*-DE-GFP reporter is activated in both cell types after injection into bovine embryos, and that the bovine OCT4-regulatory region is activated in ICM of mouse blastocysts[30]. Thus, our bovine *OCT4*-DE-GFP reporter can be activated in bovine blastocysts.

## Discussion

The bovine *OCT4* gene upstream regulatory region shares significant sequence homology with those of the humans and mice. However, despite the widespread usage of the fluorescence/luminescence reporters controlled by mouse and human *OCT4* enhancers in embryo and ESC/iPSC studies, research using bovine-specific gene reporters has been rare. Here we established lentiviral GFP-reporters controlled by bovine *OCT4* and *NANOG* enhancer regions. We showed that these reporters can be activated once infected into human and mouse ESCs, which represent primed- and naïve-state pluripotency, respectively. We further demonstrated that similar to human and mouse *OCT4*, the distal but not proximal enhancer of bovine *OCT4* is active in naïve-state pluripotent stem cells. Using the mouse iPSC induction system, we showed that these lentiviral *OCT4* reporters can serve as live indicators in iPSC induction for successful reprogramming. To our knowledge, this is the first study of bovine *OCT4* enhancer activity in different pluripotent stem cells using fluorescent reporters.

GFP-reporters controlled by *OCT4* or *NANOG* enhancer regions have greatly facilitated studies of stem cell biology and reprogramming of human and mouse cells. One interesting phenomenon found in human and mouse *OCT4* upstream regulatory sequences is the pluripotent state-specific activation for their DE and PE regions. CR4 and CR3 form part of the DE and are required for *Oct4* expression in the inner cell mass (ICM) of mouse embryos and primordial germ cells[24, 41]. Similar observations were reported for the human *OCT4* DE region in naïve-state human iPSCs[25, 26]. It has been shown that the primed-state EpiSCs are less compatible with germ-line chimera formation than naïve-state mouse ESCs[6, 8]. Also, the naïve-state human iPSCs demonstrated interspecies chimera capacity while the primed-state human ESCs could not[9]. Therefore, the generation of naive-state pluripotent bovine iPSCs will be important for study of bovine early embryo development and reproduction. Using mouse ESCs and EpiSCs, we show that as with human and mouse ESCs, the bovine *OCT4* DE region is specifically active in naïve ESCs but becomes silenced once converted to primed EpiSCs. Thus, the lentiviral GFP-reporters controlled by bovine *OCT4*-DE region can be valuable tools to help optimize and facilitate the generation and isolation of naïve-state, *bona fide* bovine iPSCs.

## Conclusion

We generated bovine specific *OCT4* and *NANOG* enhancer-controlled lentiviral GFP reporters. These reporters show activity in mouse and human ESCs, with reporters regulated by bovine *OCT4*-DE region active preferentially in naïve- but not primed-state pluripotent cells. The *OCT4*-DE region is silenced upon conversion from naïve- to primed-state, and can be reactivated when converted back from primed- to naïve-state pluripotency. Co-infection of mouse embryonic fibroblasts with the OKSM factors for iPSC induction leads to the generation of GFP+ colonies, demonstrating that these GFP reporters can serve as live indicators for induced pluripotent cell establishment. Furthurmore, the bovine *OCT4*-DE regions are active in bovine early embryos. Thus, these reporters may serve as versatile tools for completely reprogrammed bovine ESC/iPSC generation, as well as for early bovine embryo development studies.

## Supporting information

S1 Fig Schematic representation of OCT4 regulatory region upstream of the ATG codon of humans, cattle, and mice. The four conserved regions (CR1-4) among the three species, and the reported distal and proximal enhancer regions for human and mouse OCT4 are shown.
(PDF)

S1 Table Primer sequences. (PDF)
