# Supplementary material for: Analyzing bovine OCT4 and NANOG enhancer activity in pluripotent stem cells using fluorescent protein reporters
Source: PLoS One. 2018 Oct 5;13(10):e0203923. doi: 10.1371/journal.pone.0203923 (PMC6173392; doi:10.1371/journal.pone.0203923)

Analyzing Bovine OCT4 and NANOG Enhancer Activity in Pluripotent Stem Cells Using Fluorescent Protein Reporters

Authors: Delun Huang[1, 3, ¶], Ling Wang[3, ¶], Neil C. Talbot[2], Chang Huang[3], Liping Pu[1], Xiuling Zhao[1], Xiuchun Tian[3], Ming Zhang[1, *], Young Tang[3, *]

Supporting Information

S1 Fig.: Schematic representation of OCT4 regulatory region upstream of the ATG codon of humans, cattle, and mice. The four conserved regions (CR1-4) among the three species, and the reported distal and proximal enhancer regions for human and mouse OCT4 are shown.

S1 Fig.

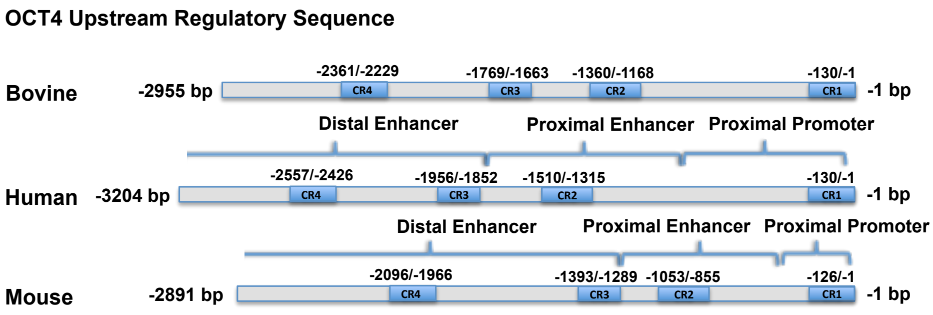

Supplement: S1 Fig — The four conserved regions (CR1-4) among the three species, and the reported distal and proximal enhancer regions for human and mouse OCT4 are shown. (PDF) [file pone.0203923.s001.pdf]
