# Supplementary material for: Analyzing bovine OCT4 and NANOG enhancer activity in pluripotent stem cells using fluorescent protein reporters
Source: PLoS One. 2018 Oct 5;13(10):e0203923. doi: 10.1371/journal.pone.0203923 (PMC6173392; doi:10.1371/journal.pone.0203923)
Supplement: S1 Table — (PDF) [file pone.0203923.s002.pdf]

S1 Table: Primer sequences.

Name	Sequence (5' – 3')
bp*Oct4*_CF1[1]	TTTTATCGATGAATTCTGATGACTCAATTGGCTTGC
bp*Oct4*_CF2[2]	TTTTATCGATGAATTCCAGTTTGCAGACCTCACAGG
bp*Oct4*_MCF[3]	TGAGCTGGAACTGGGGCTATGTGA T
bp*Oct4*_MCR[4]	CCCAGTTCCAGCTCATCTGT
bDECR1[5]	TGAGGGCTCACACTGAAGG
bDECR2[6]	TACACGCCTAACTAGTTGAGGGCTCACACTGAAGG
bPPCF1-a[7]	TCAGTGTGAGCCCTCAGAGTCTAGGAGTCTGGGGCCTG
bPECF[8]	TTTTATCGATGAATTCGGACCAGCCCAGACCCTGTG
bp*Oct4*_CR[9]	TACACGCCTAACTAGTGGGGAAGGAAGGCACCCCGA
bp*Nanog*_CF1[10]	TTTTATCGATGAATTCTGTTAGATTTCATGTTCACTGGA
bp*Nanog*_CR[11]	TACACGCCTAACTAGTGTTGCTGAGTTGAAGGAGAAGG

Note: Underlined Sequences: Fusion sequence for cloning. Products of Denoted Primers: [1,4; 3, 9]b*Oct4*-1, [2, 4; 3, 9]b*Oct4*-2, [8, 9]b*OCT4*-PE; [2, 5; 7, 9]b*OCT4*-DE2, [2, 6]b*OCT4*-DE, [10, 11]b*NANOG* (for b*OCT4*-1, b*OCT4*-2, b*OCT4*-DE2, 2 PCR fragments were ligated together for cloning)
